# Three‐Color Protein Photolithography with Green, Red, and Far‐Red Light

**DOI:** 10.1002/smll.202405687

**Published:** 2024-10-18

**Authors:** Yanjun Zheng, Fei Chen, Saskia Frank, Juan José Quispe Haro, Seraphine V. Wegner

**Affiliations:** ^1^ Institute of Physiological Chemistry and Pathobiochemistry University of Münster 48149 Münster Germany; ^2^ Xiangya School of Pharmaceutical Sciences Central South University Changsha 410083 China

**Keywords:** CarH, layer‐by‐layer (LBL) films, photolithography, photoswitchable proteins, protein micropatterning

## Abstract

Protein photolithography is an invaluable tool for generating protein microchips and regulating interactions between cells and materials. However, the absence of light‐responsive molecules that allow for the copatterning of multiple functional proteins with biocompatible visible light poses a significant challenge. Here, a new approach for photopatterning three distinct proteins on a single surface by using green, red, and far‐red light is reported. The cofactor of the green light‐sensitive protein CarH is engineered such that it also becomes sensitive to red and far‐red light. These new cofactors are shown to be compatible with two CarH‐based optogenetic tools to regulate bacterial cell‐cell adhesions and gene expression in mammalian cells with red and far‐red light. Further, by incorporating different CarH variants with varying light sensitivities in layer‐by‐layer (LbL) multiprotein films, specific layers within the films, along with other protein layers on top are precisely removed by using different colors of light, all with high spatiotemporal accuracy. Notably, with these three distinct colors of visible light, it is possible to incorporate diverse proteins under mild conditions in LbL films based on the reliable interaction between Ni^2+^‐ nitrilotriacetic acid (NTA) groups and polyhistidine‐tags (His‐tags)on the proteins and their subsequent photopatterning. This approach has potential applications spanning biofabrication, material engineering, and biotechnology.

## Graphical abstract

Three‐color protein photolithography enables the copatterning of three different proteins using green, red, and far‐red light. Here, three light‐sensitive proteins addressable with different colors of light are incorporated into layer‐by‐layer multiprotein films such that specific layers are removed upon illumination with high spatiotemporal precision, opening new possibilities in biofabrication and material engineering.

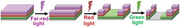

## 1 Introduction

Patterning surfaces with active proteins on a micrometer scale has enormous implications in diverse fields ranging from high‐throughput screening^[^ ^1^, ^2^ ^]^ and biosensing to protein chips,^[^ ^3^, ^4^ ^]^ tissue engineering,^[^ ^5^, ^6^, ^7^, ^8^ ^]^ control of cell‐material interactions,^[^ ^9^, ^10^ ^]^ and neuronal growth guidance.^[^ ^11^, ^12^, ^13^ ^]^ Photolithography, owing to its numerous advantages over other microfabrication techniques, has emerged as a widely favored technology. Its exceptional spatial and temporal precision, scalability, and cost‐effectiveness are the most important features in making it the preferred method of choice.^[^ ^14^, ^15^, ^16^ ^]^ Moreover, the tunability of light intensity to adjust dosage and the possibility of modulating systems without direct physical contact with the sample make light intensity an ideal tool for investigating and manipulating complex biological processes.^[^ ^17^, ^18^, ^19^ ^]^ The most prevalent techniques for photopatterning involve the use of ultraviolet (UV) light^[^ ^1^, ^4^, ^5^, ^6^, ^7^, ^9^, ^10^, ^14^, ^15^, ^17^, ^18^, ^20^, ^21^ ^]^ by incorporating photocleavable groups,^[^ ^22^, ^23^, ^24^ ^]^ azobenzenes,^[^ ^25^ ^]^ or photocrosslinking reagents^[^ ^26^, ^27^ ^]^ in protein‐anchoring groups or matrices. However, the use of UV light creates severe limitations due to its cytotoxicity to cells and the damage caused to biomolecules.

A number of studies have circumvented the use of UV light and employed visible light for protein photolithography instead.^[^ ^28^, ^29^, ^30^, ^31^, ^32^, ^33^, ^34^, ^35^, ^36^ ^]^ One possibility for creating 3D protein patterns with biocompatible near‐infrared light relies on the two‐photon absorption of existing UV light‐sensitive methods.^[^ ^37^, ^38^ ^]^ However, this process is time‐consuming due to the requirement for sequential point‐by‐point illumination. In addition, not all UV light‐sensitive molecules exhibit effective two‐photon excitation. An alternative approach, the emission of upconverting nanoparticles converting near‐infrared light into visible light, has enabled the photoactivation of light‐responsive moieties tethered to proteins.^[^ ^39^ ^]^ Another possibility for photopatterning proteins is the dimerization of tyrosines with visible light in the presence of ruthenium complexes.^[^ ^40^ ^]^ All of these examples nonetheless suffer from the inherent UV light sensitivity of the molecules and require the addition of nanoparticles or metal complexes as sensitizers, posing challenges in terms of implementation and toxicity. Moreover, the unspecific and randomly oriented immobilization of proteins may, in many cases, impair activity. Recently, light‐responsive proteins have emerged as alternative light‐sensitive entities for photopatterning proteins on surfaces and materials.^[^ ^41^, ^42^, ^43^ ^]^ Photocleavable proteins, which respond to violet to green light (400–540 nm),^[^ ^30^, ^44^, ^45^ ^]^ and photoswitchable proteins, which respond to blue (480 nm) and red/far‐red light (660–740 nm),^[^ ^46^, ^47^, ^48^ ^]^ have been used to generate protein micropatterns with biocompatible wavelengths of visible light and highly specific protein‐protein interactions. However, in these examples, the high complexity and requirements of each component prevent the copatterning of different proteins with different colors of light. Therefore, current examples of copatterned proteins are restricted to systems that use UV light and the sequential addition of proteins.^[^ ^7^ ^]^ In contrast, multicolor photopatterning of DNA with visible light has been achieved by using the photothermal effect of incorporating plasmonic nanoparticles in layer‐by‐layer films (LbL).^[^ ^49^ ^]^

Here, we report a versatile approach for the photopatterning of three different proteins with three colors of visible light. To achieve this goal, we developed new versions of the naturally green light‐responsive protein CarH with engineered cofactors, which render the proteins sensitive to red and far‐red light. These new cofactors extend the spectral response of CarH to the red and far‐red regions and open the possibility of using them for multicolor optogenetic applications in cells and multicolor protein photolithography.

## 2 Results and Discussion

### 2.1 Design of Red and Far‐Red Light‐Responsive CarH

**Figure 1:**
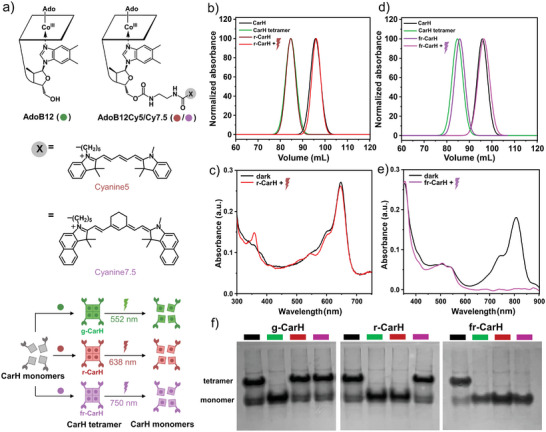
a) Structures of AdoB12, AdoB12Cy5, and AdoB12Cy7.5. CarH monomers associate with coenzymes possessing distinct photoresponsive characteristics, leading to the assembly of tetrameric structures. Upon illumination with green, red, or far‐red light, the tetramers dissociate into their respective monomers. Chromatograms of b) r‐CarH and d) fr‐CarH on a size exclusion column. The CarH monomer is shown in black, and the g‐CarH tetramer is shown in green. The r‐CarH tetramer is shown in dark red, and the fr‐CarH tetramer is shown in dark purple. r‐CarH after exposure to red light is shown in red, and fr‐CarH after exposure to far‐red light is shown in purple. UV–vis spectra showing the photoconversion of the c) r‐CarH and e) fr‐CarH tetramers (black) to the monomeric photoproduct under red (red) and far‐red (purple) illumination, respectively. f) Analysis of g‐CarH, r‐CarH, and fr‐CarH tetramers by native PAGE in the dark (black bar) or after green (green bar), red (red bar), or far‐red (purple bar) light exposure for 2 min.

CarH from *Thermus thermophilus* is a remarkable light‐responsive protein that features 5′‐deoxy adenosylcobalamin (AdoB12) as its cofactor.^[^ ^44^, ^45^ ^]^ CarH forms a tetramer in the dark in the presence of AdoB12, where histidine (H177) occupies the lower axial coordination site of the cobalamin ring. In this tetramer, green (and blue) light illumination triggers a ligand exchange reaction in which His132 displaces the adenosyl in the upper axial position, resulting in the dissociation of the CarH tetramer into its monomers. Unlike most proteins used in optogenetics, this light‐triggered reaction is irreversible in the dark or under other colors of light. This lack of reversibility is desirable in some cases, requiring sustained activation even after discontinuing light illumination, particularly for achieving stable protein patterns via photolithography. Furthermore, CarH also stands out for its unique response to blue/green light, setting it apart from the majority of proteins used in optogenetics, including LOV (light oxygen voltage) domain proteins^[^ ^50^ ^]^ and cryptochromes,^[^ ^51^ ^]^ which are reversibly activated by blue light, and phytochromes, which are typically bidirectional photoswitches that respond to red/far‐red light. To obtain a light‐sensitive protein that irreversibly responds to light from colors other than blue or green, we aimed to extend the spectral response range of CarH by engineering the cofactor AdoB12 such that it also absorbs light closer to red. Given the weak nature of the Co─C bond between adenosyl and the cobalt in the corrin ring (<30 kcal mol^−1^), we hypothesized that fluorophores excited at wavelengths surpassing the absorbance of cobalamin (>560 nm) could still convey their excited state energy to the corrin ring,^[^ ^52^, ^53^ ^]^ thereby promoting photoreaction in CarH and leading to disassembly of the tetramer into its monomers. Previous studies have shown that such fluorophores attached to AdoB12 can act as antennas, promoting the cleavage of the Co─C bond at wavelengths up to 800 nm.^[^ ^52^ ^]^ In particular, we synthesized AdoB12 conjugates with the dyes cyanine 5 (AdoB12Cy5) and cyanine 7.5 (AdoB12Cy7.5), which have absorption maxima in the red (651 nm, 44 kcal mol^−1^) and far‐red (788 nm, 36 kcal mol^−1^) regions, respectively (**Figure** **1**a).

### 2.2 Characterization of Red and Far‐Red Light‐Sensitive CarH

As the first step in validating our design, we investigated the tetramer formation of CarH in the presence of the AdoB12 derivatives with Cy5 and Cy7.5 and their ability to undergo light‐triggered disassembly (Figure 1a). To this end, we used the cobalamin binding domain of CarH from *Thermus thermophilus* (referred to as CarH throughout the article), which was expressed and purified as previously described.^[^ ^33^ ^]^ In the presence of AdoB12Cy5, CarH formed a tetramer, which eluted at a similar volume to that of the CarH tetramer formed with the native cofactor AdoB12 at 84.7 mL on a chromatogram with size exclusion chromatography (Figure 1b). After the CarH tetramer formed with AdoB12Cy5 was exposed to red light (620 nm), it dissociated into its monomers, eluting at a higher volume of 95.8 mL, similar to the starting apo‐CarH. Likewise, when CarH was incubated with AdoB12Cy7.5, a tetramer was assembled, which dissociated upon exposure to far‐red light (734 nm), as was also observed with size exclusion chromatography (Figure 1d). Hereafter, we refer to the CarH tetramers assembled in the presence of AdoB12Cy5 and AdoB12Cy7.5 as r‐CarH and fr‐CarH, respectively, in consideration of their light sensitivities. Both r‐CarH and fr‐CarH absorb within the range of their antennas, with absorption maxima at 647 and 808 nm, respectively, as observed with UV–vis spectroscopy (Figure 1c,e). For both tetramers, a change in absorption spectra was observed after exposure to the respective wavelengths, with minor changes for r‐CarH and more prominent changes for fr‐CarH. These changes are indicative of the ligand exchange reaction at the AdoB12 chromophore, and analogous changes were also observed in the case of the CarH tetramer with the native AdoB12 cofactor (g‐CarH) upon green light illumination (Figure S1, Supporting Information).

Having established r‐CarH and fr‐CarH, we evaluated the sensitivity of these different forms of CarH to various colors of light and their potential orthogonal activation. According to native polyacrylamide gel electrophoresis (native PAGE), g‐CarH exhibited a tetramer and monomer band in the dark, which migrated at different rates in the gel (Figure 1f). When g‐CarH was exposed to green light (green bar), the tetramer band completely converted to the monomer band. However, exposure to red (red bar) or far‐red (purple bar) light did not result in the loss of the tetramer band. Similarly, in the dark, r‐CarH exhibited a tetrameric band, which was converted into monomers upon exposure to green and red light but not to far‐red light. Finally, the fr‐CarH tetramer disassembled when exposed to any one of the three colors of light investigated. Overall, these findings show that the addition of the antenna extends the light absorbance range and thereby the sensitivity range with no blind spots in between.

### 2.3 Multicolor‐Controlled Optogenetics using CarH‐based Optogenetic Tools

**Figure 2:**
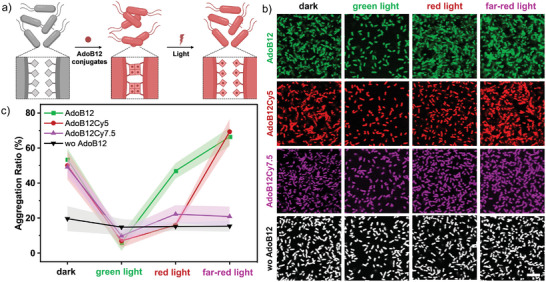
a) Schematic representation of *E. coli* MG1655 with CarH expressed at the outer membrane aggregating in the presence of AdoB12 conjugates in the dark due to CarH tetramerization. Under light (green, red, or far‐red light illumination), CarH tetramers dissociate, and bacterial aggregation is reversed. b) Fluorescence microscopy images of CarH‐displaying bacteria in the presence of different AdoB12 conjugates in the dark and under different light exposures. No aggregation of *E. coli* was found in the absence of AdoB12. Scale bar: 50 µm. c) Aggregation ratio of bacteria in b). The data are shown as the mean ± SD of three independent experiments.

Building on these findings, we explored the possibility of extending the light response range to the red region of existing optogenetic tools based on CarH. For this purpose, we evaluated two CarH‐based optogenetic tools; the green light‐sensitive bacterial cell‐cell adhesions between *Escherichia coli* ^[^ ^54^ ^]^ and the green light‐regulated gene expression in mammalian cells.^[^ ^55^ ^]^ In the former optogenetic system, bacteria express CarH on their surfaces and aggregate in the presence of AdoB12 due to CarH tetramer formation between different bacterial cells (**Figure** **2**a). As previously reported, the bacterial aggregates that formed in the presence of AdoB12 were disassembled under green light but were stable under red and far‐red light (Figure 2b). When we repeated the same experiment in the presence of AdoB12Cy5 and AdoB12Cy7.5, we observed bacterial aggregates in the dark (Figure 2b) with an aggregation ratio (area of bacteria in aggregates/total area occupied by all bacteria) of ca. 50%, as in the presence of AdoB12 (Figure 2c). This result suggested the similarly effective incorporation of different AdoB12 conjugates into CarH monomers expressed on the bacterial surface and their intercellular tetramerization. However, when these bacterial aggregates were exposed to green, red, or far‐red light, they exhibited different sensitivities. The bacterial aggregates that formed in the presence of AdoB12Cy5 were separated by exposure to either green or red light but not by exposure to far‐red light, whereas the bacterial aggregates that formed with AdoB12Cy7.5 were dispersed upon exposure to one of the three colors of light. When the bacterial aggregates disintegrated, the aggregation ratio decreased to less than 20% in all the cases, which represents the background level aggregation that was also observed in the negative control sample without any AdoB12.

**Figure 3:**
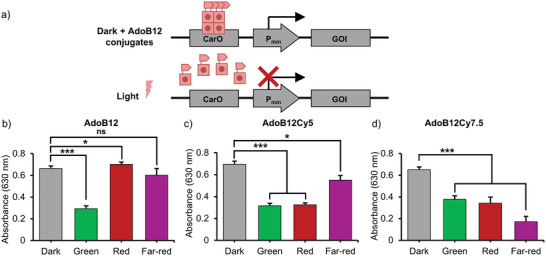
Green, red, and far‐red light‐regulated gene expression. a) In cells transfected with the CarH‐based optogenetic tool express the gene of interest (GOI) in the dark in the presence of the cofactor. On exposure to light, when the CarH tetramer dissociates, the expression of the GOI is suppressed. SEAP expression, which is here the GOI, as measured using the QUAITI‐blue assay in HeLa cells supplemented with b) AdoB12, c) AdoB12Cy5, or d) AdoB12Cy7.5.

Next, we employed the new cofactors for the photoregulation of gene expression in mammalian cells using a previously reported CarH‐based system.^[^ ^55^ ^]^ In brief, this optogenetic tool involves two constructs; the first one encoding for a CarH fused to the VP16 transactivation domain under the PSV40 promoter; the second one is a reporter plasmid with a CarO sequence upstream of the minimal human cytomegalovirus promoter sequence (Pmin), regulating the expression of secreted alkaline phosphatase (SEAP). In the dark, in the presence of AdoB12 the CarH‐VP16 tetramer forms, which binds to CarO, initiating SEAP expression. Green light exposure dissociates the CarH‐VP16 tetramer, such that it unbinds from the CarO, halting SEAP expression. Here, HeLa cells transfected with both constructs were first incubated with 10 µm of cofactors (AdoB12, AdoB12Cy5, or AdoB12Cy7.5) for 1 h in the dark and then kept for 24 h in the dark, or under green (550 nm), red (620 nm) or far‐red (734 nm) light. In the dark, SEAP expression was observed across all conditions in the presence of all three cofactors as quantified with the QUANTI‐blue assay (**Figure** **3**a–c). For cells supplemented with AdoB12, green light exposure significantly reduced SEAP production but the SEAP levels remained similar to those observed in the dark under red and far‐red light, consistent with the green light sensitivity of g‐CarH (Figure 3a). In contrast, cells supplemented with AdoB12Cy5, exhibited reduced SEAP expression under both green and red light, while far‐red light exposure did not affect SEAP production, in agreement with the light sensitivity profile r‐CarH (Figure 3b). Analogously, cells supplemented with AdoB12Cy7.5 showed reduced SEAP expression on exposure to any of the light conditions, consistent with the broad light sensitivity of fr‐CarH (Figure 3c). Importantly, cell viability was unaffected across all conditions independent of light exposure and added cofactor (Figure S2, Supporting Information). The light sensitivities observed in these cellular assays with bacteria and HeLa cells were consistent with the results of the native PAGE analysis (Figure 1f). These findings show the feasibility of using these engineered cofactors in CarH‐based optogenetic tools, extending their spectral range and suggesting the possibility of achieving multicolor responsiveness within a single system.

### 2.4 Red and Far‐Red Light Protein Photolithography

**Figure 4:**
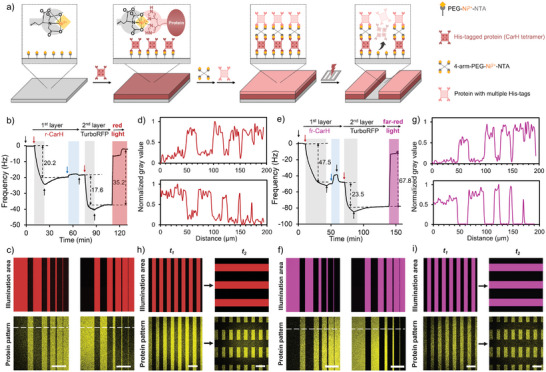
a) General strategy for the assembly of LbL multiprotein films with CarH in the first layer for red and far‐red light protein photolithography. The multi‐His6‐tagged protein in the first layer binds to the substrate through specific and multivalent interactions with Ni^2+^‐NTA groups, leaving unreacted His6‐tags on the surface. Subsequent layers were added through the reaction of the first 4‐arm‐PEG‐Ni^2+^‐NTA, and then the His6‐tagged protein of choice was added to the second layer. The light‐cleavable protein (r‐CarH or fr‐CarH) is incorporated into the first layer so that the protein in the upper layers can be removed locally by projecting a pattern of light onto the LbL multiprotein film. LbL films with b) r‐CarH or e) fr‐CarH (first layer) and TurboRFP (second layer) formed on a PEG‐Ni^2+^‐NTA‐coated SiO_2_ QCM crystal and were removed by red and far‐red light illumination, respectively. The flow of His6‐tagged proteins (5 µm r‐CarH, fr‐CarH, or TurboRFP) is shown with red arrows, four‐arm‐PEG‐Ni^2+^‐NTA (25 µm) with blue arrows, and buffer with black arrows. Illumination pattern (top) and CLSM images of TurboRFP (bottom) with a thickness of 50 to 1 µm (50, 25, 10, 5, and 1 µm) separated by 20 and 20 µm separated by a distance of 50 to 1 µm on an LbL film with c) r‐CarH or f) fr‐CarH in the first layer and TurboRFP in the second layer. TurboRFP fluorescence intensity profiles along the white lines are shown in d,g). Spatial and temporal control with illumination patterns (top) of 20 µm vertical lines projected at *t_1_* and 30 µm horizontal lines at *t_2_* and CLSM images of TurboRFP (bottom) on multiprotein films with h) r‐CarH and i) fr‐CarH in the first layer. Scale bar: 50 µm.

The second possibility for opening new versions of CarH is its use in red and far‐red light protein photolithography for generating patterns of precisely immobilized proteins and, later, in combination with multicolor photopatterning of proteins. In particular, we propose using r‐CarH and fr‐CarH as photosensitive layers in layer‐by‐layer (LbL) polyprotein films, with distinct proteins of interest in each layer. In earlier work, we developed such LbL protein films in which the first layer of the g‐CarH tetramer was immobilized through the use of four polyhistidine tags (His6‐tags) on glass surfaces with Ni^2+^‐nitrilotriacetic acid (NTA) functionalities (**Figure** **4**a).^[^ ^33^ ^]^ Here, the high specificity of the His6‐tag/Ni^2+^‐NTA interaction allows the oriented immobilization of active proteins within the LbL film. Because of the tetrameric nature of CarH in the dark and the geometry of this tetramer (dimer of dimers), multivalent interactions stabilize binding to the surface, and only two of the four His6‐tags bind to the surface. Two His6‐tags facing the solution are available for interaction with four‐arm‐polyethylene glycol (PEG)‐Ni^2+^‐NTA, which is used to bind His6‐tagged proteins in the second protein layer. The polyvalent interactions between proteins with multiple His6‐tags and four‐arm‐PEG‐Ni^2+^‐NTA thereby enabled the construction of subsequent layers in the LbL polyprotein film and the precise incorporation of distinct proteins in each layer. In this film, g‐CarH acts as a green light‐sensitive layer, and upon green light exposure, when g‐CarH dissociates into its monomers, this layer and all the layers above are released only in areas that were illuminated (Figure S3, Supporting Information).

Building on these LbL multiprotein films, we incorporated different versions of CarH, sensitive to red or far‐red light, in the first layer for subsequent protein photopatterning (Figure 4a). To validate the formation of different protein layers and the distinct light responsiveness, we monitored the LbL films by using a quartz crystal microbalance (QCM). This method enables real‐time and label‐free monitoring of macromolecule absorption and desorption on a surface, including that of proteins. Moreover, as a nonoptical method, this technique does not interfere with the light sensitivity of proteins, and the use of a window chamber allows external illumination of the surface as desired. Starting with a PEG‐Ni^2+^‐NTA‐functionalized SiO_2_ QCM crystal, a first protein layer was formed when r‐CarH was passed over it (gray background); this layer exhibited a prominent decrease in frequency and remained stable after washing with buffer (white background) (first layer: r‐CarH Δf = −20.2 Hz) (Figure 4b). The second protein layer was subsequently formed by first passing four‐arm‐PEG‐Ni^2+^‐NTA (blue background) over the QCM crystal and subsequently passing through the His6‐tagged TurboRFP (gray background), resulting in a further stable decrease in frequency after a wash with buffer (white background) (second layer TurboRFP Δf  =  −17.6 Hz). When the formed film was illuminated with red light, the frequency rapidly increased again to its initial value, revealing almost complete removal of the r‐CarH and TurboRFP layers from the surface (first and second layers total Δf = −37.8 Hz vs Δf = 35.2 Hz after red light illumination, Table S1, Supporting Information). Similarly, when the r‐CarH in the first layer was replaced with fr‐CarH, a stable protein film with multiple layers formed, and the proteins were released from the surface upon far‐red light exposure (Figure 4e, first layer fr‐CarH Δf = −47.5 Hz and second layer TurboRFP Δf = −23.5 Hz; first and second layers total Δf  =  −71.0 Hz vs Δf = 67.8 Hz after far‐red light illumination). Notably, changes in illumination cause steplike changes in frequency, and only the frequencies after the lights were turned off were used for calculating changes in frequency.

Taking advantage of the high spatiotemporal resolution that protein photolithography provides, we explored the possibility of photopatterning proteins with red or far‐red light. As described earlier, on a PEG‐Ni^2+^‐NTA‐functionalized glass slide, we formed a two‐layer protein film that included g‐CarH, r‐CarH, or fr‐CarH in the first layer and TurboRFP (fluorescent His6‐tagged protein for visualization) in the second layer as an exemplary His6‐tagged protein. Next, these surfaces were exposed to a line pattern of light for 10 s ranging from 50 to 1 µm with a spacing of 20 µm (d = 50–1 µm, s = 20 µm) by using laser wavelengths of 552 nm for g‐CarH (Figure S3c, Supporting Information) and 638 nm for r‐CarH and fr‐CarH (Figure 4c,f) for a confocal laser scanning microscope (CLSM). As a result, the TurboRFP in these illuminated areas was removed, as indicated by the dark lines contrasting the bright TurboRFP fluorescence in the unilluminated areas with a resolution down to 1 µm. Likewise, positive TurboRFP patterns were generated with down to 1 µm resolution by inverting the illumination pattern described earlier (d = 20 µm, s = 50–1 µm) (Figure 4d,g). Notably, the decrease in TurboRFP fluorescence was not due to photobleaching of TurboRFP, as surfaces coated with only Turbo‐RFP did not noticeably bleach when exposed to the same illumination conditions (Figure S4, Supporting Information). These protein patterns were modifiable over time, which we demonstrated by first generating a protein pattern with vertical lines (d = 20 µm, s = 20 µm) by using an LbL film with either r‐CarH or fr‐CarH in the first layer and TurboRFP in the second layer (Figure 4h,i, left). Subsequently, we exposed these surfaces for another 10 s to a second horizontal illumination pattern (d = 30 µm, s = 40 µm), resulting in cross‐patterns overall (Figure 4h,i, right). Overall, contingent on the specific application, these light‐sensitive LbL protein films provide versatile options for photopatterning specifically immobilized proteins with a spatial resolution down to 1 µm and a temporal resolution of 10 s with green, red, or far‐red light, overcoming problems of photodamage in UV photolithography.

### 2.5 Two‐Color Protein Photopatterning

**Figure 5:**
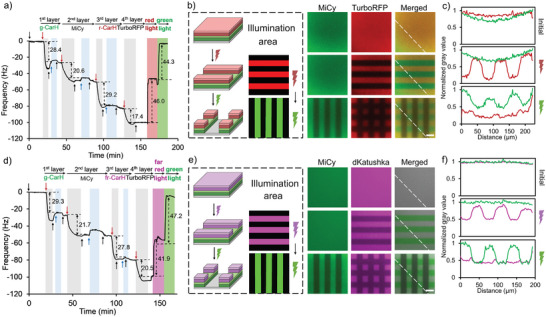
Two‐color protein photolithography. LbL films formed on the PEG‐Ni^2+^‐NTA‐coated SiO_2_ QCM crystal with g‐CarH in the first layer, MiCy in the second layer, a) r‐CarH or d) fr‐CarH in the third layer, and TurboRFP in the fourth layer; additionally, these protein layers were removed with red or far‐red light and green light. The flow of His6‐tagged proteins (5 µm) is shown with red arrows, four‐arm‐PEG‐Ni^2+^‐NTA (25 µm) with blue arrows, and buffer with black arrows. Sequential protein patterning of LbL films formed in b) and e), TurboRFP was replaced with dKatushka in the fourth layer on PEG‐Ni^2+^‐NTA‐coated glass surfaces. Fluorescence microscopy images of the surfaces of the different fluorescent proteins in the second (MiCy) and fourth (TurboRFP or dKatushka) layers showing the removal of both layers in areas exposed to green light and the removal of the fourth layer in areas exposed to red/far‐red light, as shown in c,f) in the intensity profiles along the white lines. Scale bar: 50 µm.

The distinct wavelength responses of different forms of CarH open the door for two‐color protein photolithography such that two different proteins can be copatterned on one surface. It should be noted that in these LbL films, the different layers were connected through multivalent interactions between four‐arm‐PEG‐Ni^2+^‐NTA and proteins with multiple His6‐tags used here due to their multimeric structure (CarH: tetramer, MiCy: dimer, TurboRFP: dimer, dKatushka: dimer). In particular, we prepared four‐layer protein films comprising g‐CarH in the first layer, the fluorescent His6‐tagged protein MiCy in the second layer, r‐CarH (or fr‐CarH) in the third layer, and fluorescent His6‐tagged TurboRFP (λem = 574 nm) (or dKatushka, λem = 635 nm) in the fourth layer, which emits at a different wavelength range than MiCy (λem = 495 nm). Monitoring the formation of each layer by using QCM, we observed a significant decrease in frequency following the addition of each layer, as exemplified in **Figure** **5**a, for a film with r‐CarH in the third layer (first layer g‐CarH Δf = −28.4 Hz, second layer MiCy Δf = −20.6 Hz, third layer r‐CarH Δf = −29.2 Hz, fourth layer TurboRFP Δf = −17.4 Hz). In this film, the r‐CarH in the third layer provides red light sensitivity, which, upon red light illumination, is desorbed together with the fourth layer of TurboRFP (third and fourth layers total Δf = −46.6 Hz vs Δf = 46.0 Hz after red light illumination; Table S2, Supporting Information). Moreover, the g‐CarH in the first layer and the second layer on the top were not affected by red light but were sensitive to only green light. Thus, when the film was exposed to green light, the remaining first and second layers were removed from the surface (first and second layers total Δf = −49.0 Hz vs Δf = 44.3 Hz after green light illumination; Table S2, Supporting Information). Similarly, we replaced the r‐CarH in the third layer with fr‐CarH to introduce far‐red light sensitivity to the multiprotein film and TurboRFP in the fourth layer with dKatushka, as indicated by the QCM (first layer g‐CarH Δf = −29.3 Hz, second layer MiCy Δf = −21.7 Hz, third layer fr‐CarH Δf = −27.8 Hz, fourth layer dKatushka Δf = −20.5 Hz) (Figure 5d). In this case, the third and fourth layers were removed upon far‐red light illumination (third and fourth layers total Δf = −48.3 Hz vs Δf = 41.9 Hz after far‐red light illumination; Table S2, Supporting Information); however, the first and second layers were unaffected. Green light illumination addressed the desorption of those proteins (first and second layers total Δf = −51.0 Hz vs Δf = 47.2 Hz after green light illumination; Table S2, Supporting Information), stripping the surface of all the protein layers.

These multiprotein films, which are sensitive to two different colors of light, were used for two‐color protein photolithography. Analogous to the LbL films earlier, g‐CarH and either r‐CarH or fr‐CarH were incorporated in the first and third layers, respectively, along with MiCy in the second layer and TurboRFP or dKatushka in the fourth layer. At the first time point, 50 µm wide horizontal lines (d = 50 µm, s = 50 µm) were patterned onto the four‐layer films with r‐CarH and fr‐CarH by using red and far‐red light, respectively, followed by imaging of the fluorescent proteins in the second and fourth layers (Figure 5b,e, middle). In both cases, the MiCy protein in the second layer remained homogenous across the entire surface, whereas the initially homogenous TurboRFP on top of the r‐CarH and the dKatushka on top of the fr‐CarH remained only in areas that were not exposed to red or far‐red light, respectively. At a second time point, 50 µm long vertical lines (d = 50 µm, s = 50 µm) of green light were projected on the same substrates (Figure 5b,e, bottom). Imaging of the fluorescent proteins revealed that green light illumination resulted in the dissociation of the first layer of g‐CarH, as well as the third layer of r‐/fr‐CarH, and the elimination of the connected fluorescent protein layers. The normalized gray values of the different fluorescent proteins at each step along the indicated lines further showed that with red/far‐red light, the third and fourth layers were removed from the surface with high fidelity to the illumination pattern, and with green light, all four layers were removed (Figures 4, 5). Overall, these two examples of two‐color photopatterning yield areas with no layers exposed to green light, with two protein layers exposed to red or far‐red light, and with four protein layers kept in the dark.

### 2.6 Three‐Color Protein Photolithography

**Figure 6:**
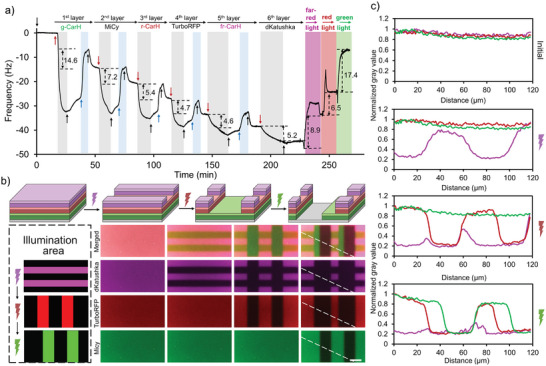
Three‐color protein photolithography. a) QCM measurements of LbL films formed on a PEG‐Ni^2+^‐NTA‐coated SiO_2_ QCM crystal with g‐CarH in the first layer, MiCy in the second layer, r‐CarH in the third layer, TurboRFP in the fourth layer, fr‐CarH in the fifth layer and dKatushka in the sixth layer and stepwise removal of these protein layers with far‐red, red, and green light. The flow of His6‐tagged proteins (5 µm) is shown with red arrows, four‐arm‐PEG‐Ni^2+^‐NTA (25 µm) with blue arrows, and buffer with black arrows. b) Three‐color protein photopatterning of LbL films formed on PEG‐Ni^2+^‐NTA‐coated glass surfaces. Fluorescence microscopy images of the surfaces of the different fluorescent proteins in the second (MiCy), fourth (TurboRFP), and sixth (dKatushka) layers showing the removal of different layers in areas exposed to green, red, and far‐red light, as shown c) in the intensity profiles along the white lines. Scale bar: 50 µm.

At last, we aimed to establish three‐color protein photolithography for the concurrent patterning of three different proteins on a multilayer protein film by using green, red, and far‐red light. To this end, we formed a six‐layer protein film with g‐CarH in the first layer (Δf = −14.6 Hz), MiCy in the second layer (Δf = −7.2 Hz), r‐CarH in the third layer (Δf = −5.4 Hz), TurboRFP in the fourth layer (Δf = −4.7 Hz), fr‐CarH in the fifth layer (Δf = −4.6 Hz), and dKatushka in the sixth layer (Δf = −5.2 Hz), as monitored by QCM (**Figure** **6**a, Table S3, Supporting Information). Upon exposure to far‐red light, the frequency increased by 8.9 Hz, indicating that the fifth and sixth layers containing fr‐CarH and dKatushka dissociated from the surface (fifth and sixth layers total Δf = −9.8 Hz). With exposure to red light, the frequency increased again by 6.5 Hz due to the detachment of the third and fourth layers containing r‐CarH and TurboRFP (third and fourth layers total Δf = −10.1 Hz). Finally, when the surface was exposed to green light, the frequency increased again by 17.4 Hz, as the first and second layers with g‐CarH and MiCy detached from the surface (first and second layers total Δf = −21.8 Hz). This stepwise and wavelength‐specific detachment of different layers indicates the possibility of copatterning up to three different proteins on the same surface. Moreover, when the same six‐layer protein film was directly exposed to green light, nearly all the proteins directly dissociated from the surface, revealing that deeper layers can be removed with light all at once (Figure S5, Supporting Information).

Encouraged by these results, we investigated the photopatterning of three different proteins by using three colors of light. Initially, the six‐layer protein film that formed on top of a Ni^2+^‐NTA‐PEG‐coated glass surface was exposed to 30 µm wide horizontal lines of far‐red light (728 nm, d = 30 µm, s = 30 µm). The initial homogenous distributions of MiCy and TurboRFP in the second and fourth layers, respectively, remained unchanged, but the dKatushka in the sixth layer was removed from areas that were illuminated with far‐red light due to the removal of fr‐CarH in the fifth layer (Figure 6b, second row). Next, the same surface was exposed to a 50 µm long red light vertical line pattern (638 nm, d = 50 µm, s = 50 µm) (Figure 6b, third row). This time, both the TurboRFP in the fourth layer and the dKatushka in the sixth layer were removed from the illuminated parts of the surface due to the disassembly of the r‐CarH and fr‐CarH in the third and fifth layers under red light, but the MiCy fluorescence in the second layer remained uniform over the surface. Finally, the surface was exposed to a 50 µm vertical green light pattern that was offset by 10 µm with respect to the previous red light pattern (d = 50 µm, s = 50 µm) (Figure 6b, fourth row). In this case, all three fluorescent proteins dKatushka, TurboRFP, and MiCy were removed from the green light‐exposed areas due to the disassembly of all three forms of the CarH tetramer. The three‐color merged image after the different steps of photopatterning, as well as the normalized gray values along the lines, shows the creation of distinct protein patterns with no fluorescence in areas exposed to green light, with only MiCy fluorescence in areas exposed to red light, with both MiCy and TurboRFP fluorescence in areas exposed to far‐red light, and with fluorescence from all three fluorescent proteins in areas that remained in the dark (Figure 6c). This multicolor photolithography procedure thereby allowed the copatterning of areas with dKatushka, TurboRFP, and MiCy in the top layer of the film alongside protein‐free areas by using three different colors of noninvasive visible light.

## 3 Conclusion

In this study, we successfully redesigned the cofactor of the green light‐sensitive protein CarH to increase its sensitivity to longer wavelengths. Unlike existing photocleavable small molecules or protein linkers, the two resultant new variants of CarH are sensitive to red and far‐red light. r‐CarH and fr‐CarH are therefore sensitive to a part of the spectrum that is highly compatible with biological molecules and has high tissue penetration potential for in vivo applications. On the one hand, for applications in photopatterning and materials, red/far‐red light sensitivity completely circumvents the obstacles that arise from the use of two‐photon activation and upconverting nanoparticles; r‐CarH and fr‐CarH can thus be implemented as new light‐sensitive elements. On the other hand, in the field of optogenetics, r‐CarH and fr‐CarH expand the existing toolkit with the unique possibility of irreversibly dissociating proteins with red and far‐red light. Notably, this is the first example of cofactor engineering of a light‐sensitive protein to alter its light sensitivity. As demonstrated in this study for CarH‐based bacterial cell‐cell adhesions, regulation of gene expression, and protein photopatterning on LbL CarH protein films, these new cofactors can be also used in combination with other existing CarH‐based optogenetic^[^ ^56^, ^57^ ^]^ tools and materials^[^ ^58^, ^59^ ^]^ to confer sensitivity to red and far‐red light.

It should be noted that handling r‐CarH and fr‐CarH presents technical challenges due to the irreversible photolysis of the cofactor with different wavelengths of light across the visible spectrum. All samples must be handled with great care to avoid light exposure, and the greater the absorbance shift toward the red, the more challenging this becomes. Similarly, selecting fluorescent probes for visualizing protein patterns requires careful consideration. Where possible, fluorophores that are excited outside the absorption range of the CarH protein should be used. If this is not feasible, only brief light exposure in the absorption range is tolerated only minimally affecting the system. In this case, for real‐time analysis, non‐optical methods are preferable (e.g., QCM in this study) or endpoint measurements should be conducted.

We particularly focused on protein photolithography, in which we achieved two major advances. First, the method that we developed allows photopatterning of any His6‐tagged protein of choice with red and far‐red light. The use of His6‐tagged proteins is generally applicable because most recombinantly produced proteins are purified from their source organism through the addition of His6‐tags. This approach overcomes the requirement of fusing proteins of interest to specific light‐sensitive protein domains,^[^ ^47^ ^]^ as is the case in studies that rely on photoswitchable proteins for protein photolithography. Moreover, the specific binding between the His6‐tag and the connecting four‐arm‐PEG‐Ni^2+^‐NTA groups assures oriented and gentle protein immobilization, resulting in better maintenance of protein activity. Second, the strategic combination of CarH tetramers with different sensitivities to light in multiprotein films enables the copatterning of up to three different proteins on the same surface by using green, red, and far‐red light. Notably, proteins that are placed in the intermediate layers of these LbL films must have multiple His6‐tags to form polyvalent interactions that connect to the layers below and above. In contrast to other reports of copatterning proteins, in the present study, proteins were removed from the surfaces upon illumination and not added to different parts of the surface sequentially by activating reactive groups on the surface. Although prevailing techniques permit the patterning of functional proteins by using visible light, none have demonstrated the ability to manipulate functional proteins with red or far‐red light while concurrently copatterning multiple proteins. Overall, red and far‐red light‐sensitive CarHs expand the possibilities for light‐responsive materials and optogenetics and introduce new avenues for protein photolithography, photoregulation of cellular behavior, and engineering of cell‐material interactions.

## 4 Experimental Section

### Materials

The proteins MiCy (Addgene plasmid # 54565), TurboRFP (Addgene plasmid # 54858), and dKatushka (Addgene plasmid # 54775) were acquired from Addgene. The coding sequence for CarH was inserted into the vector pET‐22b between the NdeI and BamHI cutting sites. BL21 (DE3) *E. coli* was purchased from New England Biolabs. Four‐arm‐PEG‐succinimidyl NHS ester (MW 10 kDa) was purchased from Creative PEG Works. A Ni^2+^‐NTA column (HisTrap^TM^ HP, column volume 5 mL) was purchased from GE Healthcare Life Sciences. A high‐performance liquid chromatography (HPLC) column (Luna 5 µm C18(2) 100 Å 250 × 10 mm reversed phase) was purchased from Phenomenex. All the organic solvents (acetonitrile (CH_3_CN), chloroform (CHCl_3_), dichloromethane (DCM), dimethylformamide (DMF), dimethyl sulfoxide (DMSO), and methanol (MeOH)) were obtained from Fisher Scientific and used without further purification (HPLC or analytical grade). All the other chemicals were purchased from Sigma‒Aldrich/Merck. Cyanine5 NHS ester and Cyanine7.5 NHS ester were purchased from Lumiprobe.

### Synthesis of AdoB_12_‐Fluorophore Conjugates

The synthesis of the AdoB_12_‐ethylenediamine conjugate followed a previously reported method.^[^ ^52^ ^]^ In brief, AdoB_12_ (0.0209 g, 13 µmol) and 1,1΄‐carbonyl‐di‐(1,2,4‐triazole) (0.0142 g, 87 µmol) were dissolved in 0.2 mL of dry DMF and stirred at room temperature for 1 h under a N_2_ atmosphere. Subsequently, ethylenediamine (0.0270 g, 450 µmol) was added to the reaction mixture, and the mixture was stirred for an additional 18 h. The resultant compound was purified using an HPLC C‐18 column with a linear gradient of the binary solvent system (solvent A: 0.1% TFA/H_2_O; solvent B: 0.1% TFA/CH_3_CN) at varying ratios of A:B from 97:3 to 10:90 over 120 min at a flow rate of 1 mL min^−1^. The removal of solvent through lyophilization resulted in the formation of an orange solid (0.0171 g, yield: 77.4%).

### Synthesis of AdoB_12_‐Fluorophore Conjugates: AdoB12Cy5 and AdoB12Cy7.5

The synthesis of the AdoB12‐fluorophore conjugates followed a previously reported method.^[^ ^52^ ^]^ Specifically, the N‐hydroxysuccinimide esters of the fluorophores Cy5 or Cy7.5 (1 eq.) were mixed with AdoB_12_‐ethylenediamine conjugate (1.5 eq.) and diisopropylethylamine (6 eq.) in 2 mL of dry DMF and stirred for 18 h. Subsequently, the reaction mixture was purified using an HPLC C‐18 column with a linear gradient of a binary solvent system (solvent A: 0.1% TFA/H_2_O; solvent B: 0.1% TFA/CH_3_CN) by varying the ratio of A:B from 97:3 to 10:90 over 120 min at a flow rate of 1 mL min^−1^. The dark blue product (AdoB12Cy5), with peaks at both 360 and 650 nm, and the dark green product (AdoB12Cy7.5), with absorption at both 360 and 780 nm, were collected. The removal of solvent through lyophilization resulted in the formation of blue and green solid products, respectively.

### Protein Purification

For protein production, each protein expression plasmid was transformed into BL21(DE3) *E. coli*, which were subsequently plated on an LB‐agar plate supplemented with ampicillin (50 µg mL^−1^) and grown overnight at 37 °C. A single colony was inoculated into 10 mL of LB medium supplemented with ampicillin (50 µg mL^−1^) and incubated at 37 °C with shaking at 250 rpm overnight. The overnight culture was transferred to 1 L of LB medium supplemented with ampicillin (50 µg mL^−1^) and incubated at 37 °C and 250 rpm until an OD_600_ of 0.6–0.8 was reached. Protein expression was induced by the addition of 1 mm isopropyl‐β‐D‐thiogalactopyranoside (IPTG) to CarH and 1 g L^−1^ L‐(+)‐arabinose to MiCy, TurboRFP, and dKatushka, and the bacteria were cultured at 18 °C with shaking at 250 rpm overnight. The next day, the cells were harvested by centrifugation (6000 rpm, 4 °C, 8 min) (Beckman Coulter Avanti J‐26S XP, JA‐10 rotor), the supernatant was discarded, and the bacterial pellet was resuspended in 20 mL of lysis buffer (50 mm Tris‐HCl, 300 mm NaCl, pH 7.4) supplemented with 1 mm protease inhibitor phenylmethane sulfonyl fluoride (PMSF) and 1 mm DL‐dithiothreitol (DTT). The bacteria were lysed by sonication, and the lysate was separated by centrifugation at 12 000 rpm and 4 °C for 30 min (Beckman Coulter Avanti J‐26S XP, JA‐25.50 rotor), after which the supernatant was filtered through a 0.45 µm filter twice. The clarified lysate was loaded onto a 5 mL Ni^2+^‐NTA agarose column. After washing with 50 mL of buffer (50 mm Tris‐HCl, 300 mm NaCl, 25 mm imidazole, and 1 mm DTT, pH 7.4), the protein was eluted with 10 mL of buffer (50 mm Tris‐HCl, 300 mm NaCl, 250 mm imidazole and 1 mm DTT, pH 7.4). The purified proteins were dialyzed against 2 L of buffer (50 mm Tris‐HCl, 300 mm NaCl, 1 mm DTT, pH 7.4) twice for 6 h each.

### Preparation of Green, Red, and Far‐Red Light‐Responsive CarH Tetramers (g‐CarH, r‐CarH and fr‐CarH)

The purified CarH monomer (100 µm) was incubated with a fivefold molar excess of AdoB_12_, AdoB12Cy5, or AdoB12Cy7.5 overnight in the dark at 4 °C to form the CarH tetramer. Unbound AdoB_12_ was removed from the resulting CarH tetramer by size exclusion chromatography (SEC) using a HiLoad^TM^ 16/600, Superdex^TM^ 200 pg size exclusion column in the dark. The CarH tetramer was concentrated using centrifugal filtration devices (10 kDa molecular weight cutoff) if necessary.

### Native PAGE

To analyze the dissociation behavior of each CarH tetramer, native PAGE was performed. Prior to PAGE, samples of g‐CarH, r‐CarH, or fr‐CarH were kept in the dark under green (550 nm, 540 µW cm^−2^), red (620 nm, 1440 µW cm^−2^), or far‐red (734 nm, 1120 µW cm^−2^) LED light illumination, respectively, for 2 min. Each protein sample was then mixed with 2x sample buffer (62.5 mm Tris‐HCl (pH 6.8), 40% (w/v) glycerol, and 0.01% (w/v) bromophenol blue) at a 1:1 ratio. Five microliters of protein sample were loaded onto a 10% PAGE gel (formulation: 4.11 mL of water, 3.33 mL of 30% acrylamide/bis‐acrylamide 29:1, 2.5 mL of 1.5 m Tris‐HCl (pH 8.8), 60 µL of 10% APS (ammonium persulfate), and 10 µL of TEMED (tetramethylethylendiamin) with a 4% stacking gel (6.2 mL of water, 2.5 mL of 30% acrylamide/bis‐acrylamide 29:1, 1.33 mL of 0.5 m Tris‐HCl (pH 6.8), 60 µL of 10% APS, and 15 µL of TEMED) on top. The gel electrophoresis was performed at room temperature in the dark at a constant voltage of 80 V in running buffer (0.025 m Tris, 0.192 m glycine, pH 8.2–8.4). After 10 min, the voltage was increased to 125 V for 1 h. The gel was stained using a standard Coomassie blue staining protocol.

### Bacterial Aggregation

For bacterial aggregation experiments, *E. coli* MG1655 cells transformed with CarH‐eCPX and pTRC99a‐mCherry as reported previously were used.^[^ ^54^ ^]^ Overnight cultures of *E. coli* transformed with CarH‐eCPX were harvested through centrifugation, followed by washing with PBS. Subsequently, the cultures were diluted to an optical density at 600 nm (OD600) of 0.1 (≈1 × 10^8^ cells mL^−1^). Volumes of 300 µL of bacterial suspension were dispensed into individual wells of four eight‐well ibidi µ‐slide dishes, each supplemented with 500 µM AdoB12, AdoB12Cy5, or AdoB12Cy7.5. The samples were incubated under static conditions for 3 h at room temperature either in darkness (within an opaque container or fully wrapped in aluminum foil) or under green (550 nm, 540 µW cm^−2^), red (620 nm, 1440 µW cm^−2^), or far‐red (734 nm, 1120 µW cm^−2^) LED light illumination. Subsequently, CLSM images (Leica TCS SP8, equipped with a 552 nm and a 40× objective) were acquired for an area of 2 × 2 mm at the center of each well. Image analysis was performed using an ImageJ particle analyzer, where the aggregation ratio was defined as the area occupied by bacterial clusters (objects with an area > 25 µm2, equivalent to the size of approximately ten individual bacteria) divided by the total area encompassing all bacteria. The results were reported as the average of three independent experiments with three technical replicates per experiment.

### Photoregulated Gene Expression

Human cervix carcinoma cells (HeLa, ATCC CCL‐2) were cultivated in DMEM medium (Dulbecco's modified Eagle's medium, Gibco, cat# 10565018) supplemented with 10% FBS (fetal bovine serum, Sigma Aldrich, F2442) and 1% P/S (penicillin/streptomycin, Jena BioScience, ML‐105XL) at 37 °C and 5% CO_2_. Cells were transfected with CarH‐VP16 (pHB144) and pCVC036 plasmids (kindly provided by Prof. Wilfried Weber) in a 4:1 ratio (w:w) using lipofectamine 3000 (Thermo Fisher Scientific, L300001), following the manufacturer's protocol for a 24‐well plate. After 24 h, the medium was replaced with fresh medium supplemented with 10 µm AdoB12, AdoB12Cy5, or AdoB12Cy7.5 as cofactors. All experimental procedures after the addition of the cofactors were carried out in the dark. After 1 h incubation in the dark, the cells were kept in the dark wrapped in aluminum foil or illuminated with green (550 nm, 540 µW cm^−2^), red (620 nm, 1440 µW cm^−2^) or far‐red light (734 nm, 1120 µW cm^−2^) for 24 h. The secreted alkaline phosphatase (SEAP) activity was measured by combining 20 µL of supernatant with 180 µL Quanti‐Blue SEAP detection reagent (InvivoGen) in a 96‐well assay plate. The samples were incubated at 37 °C and 5% CO_2_ for 1 h and absorbance at 630 nm was measured using a multimode plate reader (Spark, Tecan Life Science).

To test for potential light toxicity, 1 × 10^4^ cells per well were seeded in 100 µL medium into 96‐well microplates and exposed to the different wavelengths of light in the presence of 10 µm of the different cofactors for 1 h as in the corresponding experiment. Afterward, 10 µL of 5 mg mL^−1^ MTT was added to each well and the cells were incubated for 3 h under standard culture conditions. Then, 100 µL of DMSO was added to each well to solubilize the formazan crystals and the absorbance at 570 nm was measured using a multimode plate reader. The viability of cells without any treatment was set to 100%.

### Preparation of Four‐Arm‐PEG‐NTA

Four‐arm‐PEG‐NTA was synthesized as previously reported.^[^ ^33^ ^]^ *N_α_,N_α_*‐Bis(carboxymethyl)‐L‐lysine hydrate (NTA‐Lys) (0.24 mmol, 65 mg) was first dissolved in 10 mL of MeOH. Subsequently, four‐arm‐PEG N‐hydroxysuccinimide ester (four‐arm‐PEG‐NHS, MW 10 kDa) (0.06 mmol, 600 mg) was added to the mixture, which was subsequently stirred until dissolved. Finally, 60 µL of four‐methylmorpholine was added to the solution as a catalyst. After reacting for 6 h at room temperature, the product was precipitated by adding diethyl ether (100 mL). The product was filtered and dried under a vacuum.

### Glass Surface Functionalized with PEG‐Ni^2+^‐NTA

The glass surfaces were functionalized similarly to previous methods.^[^ ^46^ ^]^ In brief, glass slides (20 × 20 mm) were cleaned with freshly prepared piranha solution (3:1 (*v/v*) concentrated H_2_SO_4_:H_2_O_2_ (30%)) for 1 h, rinsed 3 times with Milli‐Q water and dried with a N_2_ stream. For the PEGylation reaction, the surfaces were immersed in a solution of PEG_3000_‐azide (10 mg of PEG_3000_‐azide, MW = 3500 g mol^−1^) and 200 µL of dry triethylamine in dry toluene (50 mL) and kept at 80 °C overnight under a N_2_ atmosphere. The next day, the surfaces were first washed with ethyl acetate for 5 min by sonication, then with methanol for 5 min by sonication, and subsequently dried with a N_2_ stream. The PEG‐coated surfaces were incubated with 100 µL of reaction solution containing 100 mm *L*‐ascorbic acid, 100 mm Tris‐HCl (pH 9.0), 150 µm NTA‐alkyne and 1 mm CuSO_4_ in a moist chamber for 2 h at room temperature. The surfaces were incubated with the following solutions to obtain PEG‐Ni^2+^‐NTA‐functionalized surfaces: 1) 50 mm EDTA (pH 7.4) for 5 min; 2) buffer (50 mm Tris‐HCl, 300 mm NaCl, pH 7.4) twice for 5 min; 3) 0.1 m NiCl_2_ in Milli‐Q water for 5 min; and 4) buffer (50 mm Tris‐HCl, 300 mm NaCl, pH 7.4) for 5 min.

### Protein Immobilization on PEG‐Ni^2+^‐NTA‐Functionalized Glass Surfaces

To obtain protein‐functionalized layer‐by‐layer (LbL) surfaces, the surfaces were incubated with 100 µL of the following solutions and washed with buffer (50 mm Tris‐HCl, 300 mm NaCl, pH 7.4).

One‐color protein photolithography was performed as follows: 1) first layer: 5 µm purified g‐CarH, r‐CarH, or fr‐CarH for 30 min; 2) buffer for 10 min; 3) 25 µm four‐arm‐PEG‐NTA + 100 µm NiCl_2_ in buffer for 30 min; 4) buffer for 10 min; and 5) 5 µm TurboRFP for 30 min (second fluorescent protein layer for imaging). 6) Buffer for 10 min.

Two‐color protein photolithography was performed as follows: 1) first layer: 5 µm purified g‐CarH for 30 min; 2) buffer for 10 min; 3) 25 µm 4‐arm‐PEG‐NTA + 100 µm NiCl_2_ in buffer for 30 min; 4) buffer for 10 min; and 5) 5 µm MiCy for 30 min (second fluorescent protein layer for imaging). 6) Buffer for 10 min; 7) 25 µm 4‐arm‐PEG‐NTA + 100 µm NiCl_2_ in buffer for 30 min; 8) buffer for 10 min; 9) 5 µm purified r‐CarH or fr‐CarH for 30 min; 10) buffer for 5 min; 11) 25 µm 4‐arm‐PEG‐NTA + 100 µm NiCl_2_ in buffer for 30 min; 12) buffer for 5 min; 13) 5 µm TurboRFP or dKatushka for 30 min; 14) buffer for 10 min.

Three‐color protein photolithography was performed as follows: 1) first layer: 5 µm purified g‐CarH for 30 min; 2) buffer for 10 min; 3) 25 µm 4‐arm‐PEG‐NTA + 100 µm NiCl_2_ in buffer for 30 min; 4) buffer for 10 min; and 5) 5 µm MiCy for 30 min (second fluorescent protein layer for imaging). 6) Buffer for 10 min. 7) 25 µm 4‐arm‐PEG‐NTA + 100 µm NiCl_2_ in buffer for 30 min. 8) Buffer for 10 min. 9) 5 µm purified r‐CarH for 30 min. 10) Buffer for 5 min. 11) 25 µm 4‐arm‐PEG‐NTA + 100 µm NiCl_2_ in buffer for 30 min. 12) Buffer for 5 min. 13) 5 µm TurboRFP for 30 min (fourth fluorescent protein layer for imaging). 14) Buffer for 10 min. 15) 25 µm 4‐arm‐PEG‐NTA + 100 µm NiCl_2_ in buffer for 30 min. 16) Buffer for 10 min. 17) fr‐CarH for 30 min. 18) Buffer for 10 min. 19) 25 µm 4‐arm‐PEG‐NTA + 100 µm NiCl_2_ in buffer for 30 min. 20) Buffer for 10 min. 21) 5 µm dKatushka for 30 min (sixth fluorescent protein layer for imaging). 22) Buffer for 10 min.

### Protein Patterning

For one‐ and two‐color protein photolithography, straight‐line illumination patterns were produced via confocal laser scanning microscopy (Leica TCS SP8) instrument equipped with an argon laser and 40x H_2_O objective. For one‐color protein photolithography, straight lines were projected onto the LbL film using a 50% intensity 552 nm (10 mW) for g‐CarH, 638 nm (15 mW) for r‐CarH, or 638 nm (15 mW) for fr‐CarH laser for 10 s. For two‐color protein photolithography, straight lines were projected onto the LbL film using 50% intensity of the 638 nm (10 mW) for 10 s of illumination first and then 50% intensity of the 552 nm (10 mW) for 10 s of illumination. For three‐color protein photolithography, line patterns of proteins were produced using a confocal laser scanning microscope equiped with a white light laser (Leica Stellaris) and a 60x H_2_O objective. Initially, transverse lines were projected onto the LbL film using a 728 nm excitation line for 10 s. Subsequently, longitudinal lines were projected onto the LbL film for 10 s using a 638 nm excitation line. Finally, intersecting lines were projected onto the LbL film for 10 s using a 552 nm excitation line.

### QCM Measurement for the LbL System

All QCM measurements were performed on a Q‐Sense E4 system (Q‐Sense) with SiO_2_ crystals (Q‐sense) at room temperature with a flow rate of 80 µL min^−1^. First, the SiO_2_ crystals were cleaned by sonication in 2% sodium dodecyl sulfate (SDS) solution for 30 min, copiously rinsed with Milli‐Q water, and subsequently dried in a stream of N_2_. Next, the QCM crystals were activated by ultraviolet ozone (UV‐ozone) for 10 min (Ossila, L2002A2‐UK) and functionalized with PEG‐Ni^2+^‐NTA as described above for glass surfaces. The treated crystals were placed into QCM chambers, and the following solutions were passed over the crystals to form LbL protein films: 1) buffer, 5 min; 2) 5 µm first protein, 30 min; 3) buffer, 10 min; 4) 100 µm NiCl_2_ + 25 µm 4‐arm‐PEG‐NTA, 30 min; 5) buffer, 5 min; 6) 5 µm second protein, 30 min; and 7) buffer, 10 min. Steps 4–7 were repeated to form additional protein layers. Experiments that included green, red, and far‐red light illumination were performed with a window QCM module, and green (550 nm, 540 µW cm^−2^), red (620 nm, 1440 µW cm^−2^), or far‐red (734 nm, 1120 µW cm^−2^) LED lamps were used for illumination from above.

## Conflict of Interest

The authors declare no conflict of interest.

## Author Contributions

Y.Z., F.C., and S.V.W. designed the experiments. Y.Z. performed the experiments with support from FC, analyzed the data, and prepared the figures. Y.Z. conducted the native PAGE experiment under the guidance of S.F., and J.J.Q.H. conducted the bacterial aggregation experiments. All the authors read and reviewed the results and approved the final version of the manuscript.

## Supporting information



Supporting Information

## Data Availability

The data that support the findings of this study are available from the corresponding author upon reasonable request.
