# Supplementary material for: Three‐Color Protein Photolithography with Green, Red, and Far‐Red Light
Source: Small. 2024 Oct 18;20(52):2405687. doi: 10.1002/smll.202405687 (PMC11673460; doi:10.1002/smll.202405687)
Supplement: Supplementary file 1 — Supporting Information [file SMLL-20-2405687-s001.pdf]

NANO · MICRO
small

Supporting Information

for *Small*, DOI 10.1002/smll.202405687

Three-Color Protein Photolithography with Green, Red, and Far-Red Light

*Yanjun Zheng, Fei Chen, Saskia Frank, Juan José Quispe Haro and Seraphine V. Wegner**

Supporting information

Three color protein photolithography with green, red and far-red light

Yanjun Zheng, [1§] *Fei Chen,*[1-2§] *Saskia Frank,*[1] *Juan José Quispe Haro,*[1]*Seraphine V. Wegner*[1*]

[1] Institute of Physiological Chemistry and Pathobiochemistry, University of Münster, 48149 Münster, Germany

[2] Xiangya School of Pharmaceutical Sciences, Central South University, 410013 Changsha, P. R. China

[§]These authors contributed equally.

*Corresponding authors: wegnerse@uni-muenster.de (S. V. W.)

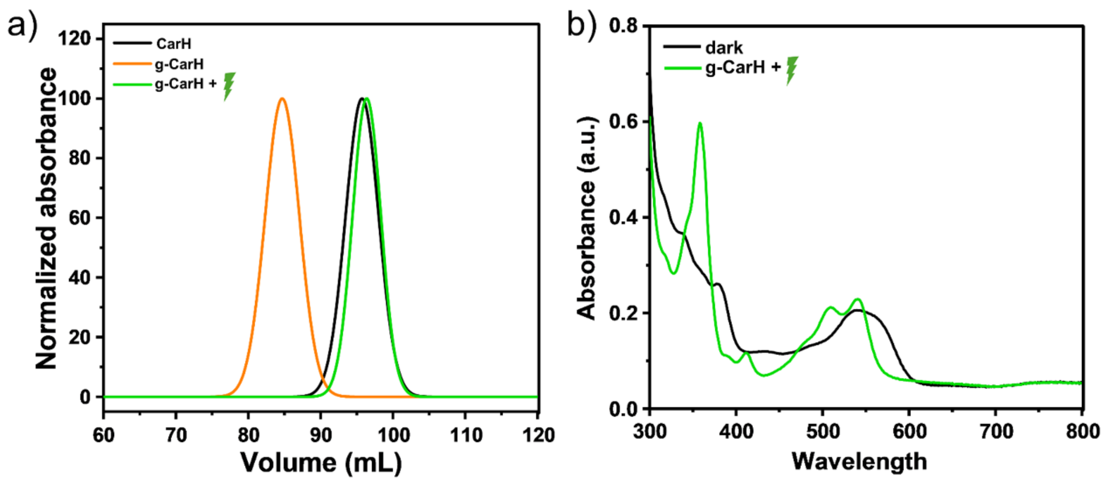

Figure S1. a) Chromatogram of g-CarH on a size exclusion column. Orange spectra: g-CarH tetramer. Black spectra: CarH monomer. Under green light illumination, CarH tetramer dissociates into its monomers (green spectra). b) Ultraviolet-visible spectra showing the photoconversion of CarH tetramer (black spectra) to the monomeric photoproduct (green spectra) after green light illumination.

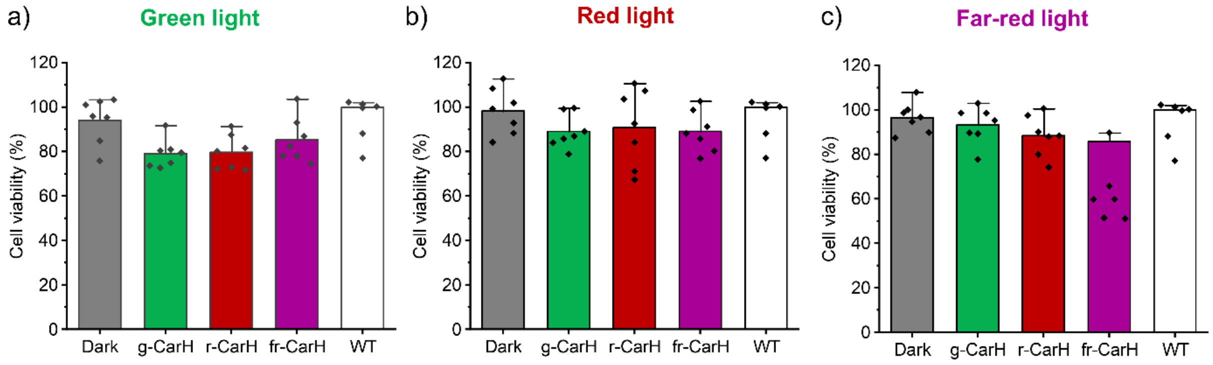

Figure S2. Cell viability under a) green, b) red and far-red light in the presence of different co-fators as measured using the MTT assay.

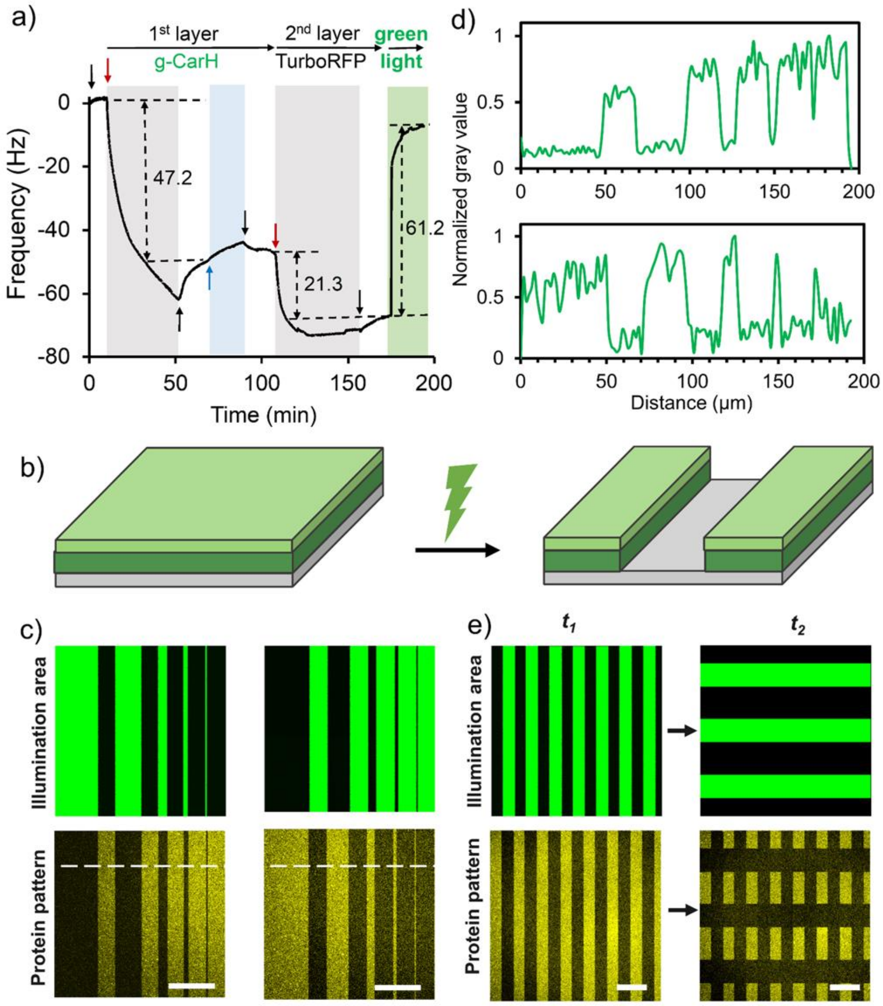

Figure S3. A light-sensitive LbL protein film using the green light cleavable protein. a) g-CarH A (1st layer) and TurboRFP (2nd layer) are formed on a PEG-Ni^{2+}-NTA coated SiO_2 QCM crystal and removed upon green light illumination. Red arrows: g-CarH or His-tagged TurboRFP. blue arrows: 4-arm-PEG-Ni^{2+}-NTA, black arrows: buffer. The concentration of each solution: 5 μM of the respective protein, 25 mM 4-arm-PEG-Ni^{2+}-NTA. b) Schematic images of green light photolithography. c), d) Protein patterns with a thickness of 50 μm to1 μm (50 μm, 25 μm, 10 μm, 5 μm and 1 μm) separated by 20 μm, and 20 μm protein patterns separated by distance from 50 μm to1 μm on an LbL film with g-CarH (1st layer) and TurboRFP (2nd layer). e) Spatial and temporal control over protein patterns using green light photolithography. Firstly, 20 μm vertical lines were patterned onto an LbL CarH/TurboRFP film (t_1). Subsequently, 30 μm horizontal lines were patterned onto the same substrate (t_2), resulting in a cross pattern. Top: Projected light pattern, bottom: obtained protein pattern. Scale bar: 50 μm.

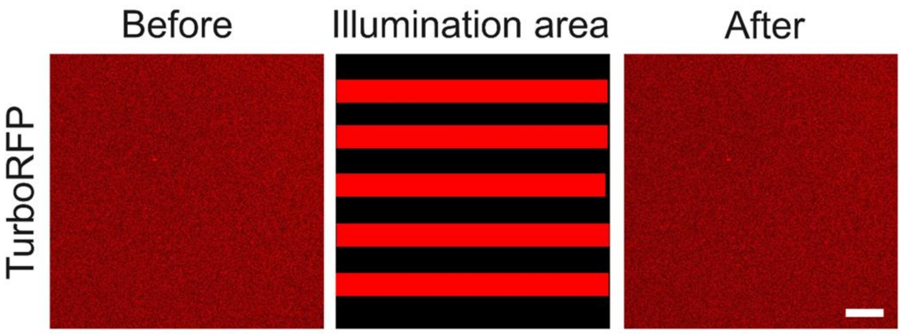

Figure S4. A protein film using the TurboRFP protein. TurboRFP is formed on PEG-Ni^{2+}-NTA coated glass slides and with no notable protein bleaching after 10s light exposure in a line pattern observed. Scale bar: 50 μm.

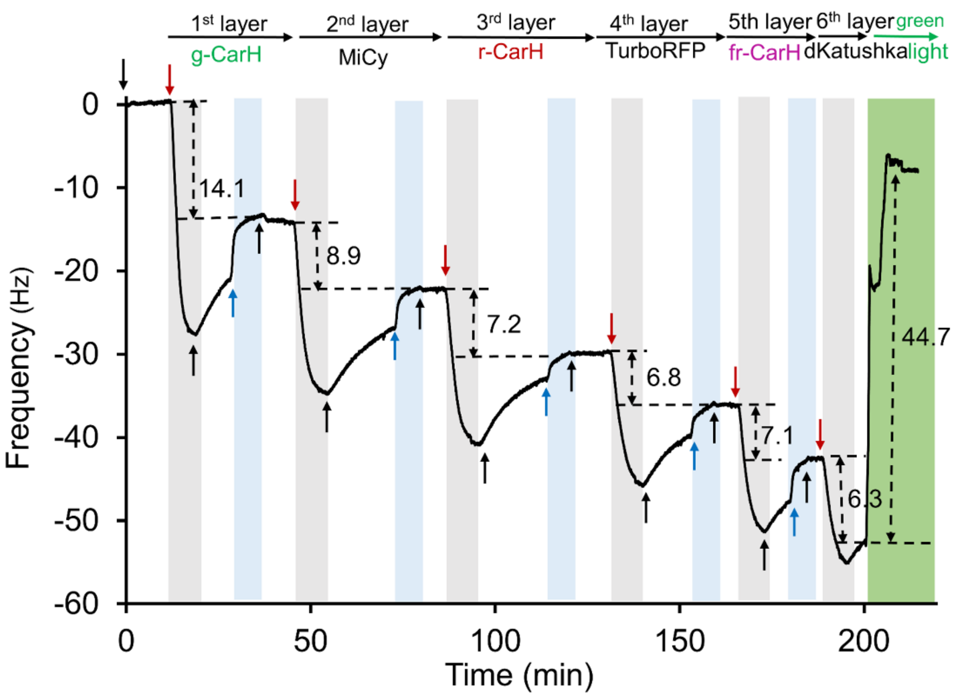

Figure S5. QCM measurement of multicolor photolithography. A light-sensitive LbL protein film using the a) green (1st layer g-CarH) + red (3rd layer r-CarH) + far-red (5th layer fr-CarH) light cleavable protein and fluorescent His-tagged protein (MiCy in 2nd, TurboRFP in 4th and dKatushka in 6th layer) is formed on a PEG-Ni^{2+}-NTA coated SiO_2 QCM crystal and removed upon green light illumination. Red arrows: g-CarH, r-CarH, fr-CarH, or fluorescent His-tagged protein, blue arrows: 4-arm-PEG-Ni^{2+}-NTA, black arrows: buffer. The concentration of each solution: 5 µM of the respective protein, 25 mM 4-arm-PEG-Ni^{2+}-NTA.

Table S1

	Protein	ΔF	Protein	ΔF	Protein	ΔF
1st layer	g-CarH	47.2	r-CarH	20.2	fr-CarH	47.5
2nd layer	Turbo RFP	21.3	Turbo RFP	17.6	Turbo RFP	23.5
**Total**		**68.5**		**37.8**		**71**
	**green light**	61.2	**red light**	35.2	**far-red light**	67.8

- Frequency shift (ΔF)

Table S1. Frequency shifts during protein adsorption and dissociation in QCM measurements of single photoresponsive LbL protein films.

Table S2

	Protein	ΔF	Protein	ΔF
1st layer	g-CarH	28.4	g-CarH	29.3
2nd layer	MiCy	20.6	MiCy	21.7
**Total**		**49.0**		**51.0**
	**green light**	44.3	**green light**	47.2
3rd layer	r-CarH	29.2	fr-CarH	27.8
4th layer	Turbo RFP	17.4	dKatushka	20.5
**Total**		**46.6**		**48.3**
	**red light**	46.0	**far-red light**	41.9

- Frequency shift (ΔF)

Table S2. Frequency changes during protein adsorption and dissociation in QCM measurements of two-color photolithographic protein films.

Table S3

	Protein	ΔF
1st layer	g-CarH	14.6
2nd layer	MiCy	7.2
**Total**		**21.8**
	green light	17.4
3rd layer	r-CarH	5.4
4th layer	Turbo RFP	4.7
**Total**		**10.1**
	red light	6.5
5th layer	fr-CarH	4.6
6th layer	dKatushka	5.2
**Total**		**9.8**
	far-red light	8.9

- Frequency shift (ΔF)

Table S3. Frequency changes during protein adsorption and dissociation in QCM measurements of three-color photolithographic protein films.

Table S4

	Protein	ΔF
1st layer	g-CarH	14.1
2nd layer	MiCy	8.9
3rd layer	r-CarH	7.2
4th layer	Turbo RFP	6.8
5th layer	fr-CarH	7.1
6th layer	dKatushka	6.3
**Total**		**50.4**
	green light	44.7

- Frequency shift (ΔF)

Table S4. Frequency shifts observed during protein adsorption and dissociation in QCM measurements of three-color photolithographic protein films under green light illumination.

Table S5

Name	DNA Sequence
**Micy-pBAD**	TCGCAACTCTCTACTGTTTCTCCATACCCGTTTTTTTGGGCTAGAAATAATTTTGTTTA<br>ACTTTAAGAAGGAGATATACATATGCGGGGTTCTCATCATCATCATCATCATGGTAT<br>GGCTAGCATGACTGGTGGACAGCAAATGGGTCGGGATCTGTACGAGAACCTGTACT<br>TCCAGGGCTCGAGCATGGTGTCTTATTCAAAGCAAGGCATCGCACAAGAAATGCGG<br>ACGAAATACCGTATGGAAGGCAGTGTCAATGGCCATGAGTTCACGATCGAAGGTGT<br>AGGAACTGGAAACCCTTACGAAGGGAAACAGATGTCCGAATTAGTGATCATCAAGC<br>CTAAGGGAAAACCCCTTCCATTCTCCTTTGACATACTGTCAACAGCCTTTCAATATG<br>GAAACAGATGCTTCACAAAGTACCCTGCCGACATGCCTGACTATTTCAAGCAAGCAT<br>TCCCAGATGGAATGTCATATGAAAGGTCATTTCTATTTGAGGATGGAGGAGTTGCTA<br>CAGCCAGCTGGAGCATTCGTCTCGAAGGAAATTGCTTCATCCACAATTCCATCTATC<br>ATGGCGTAAACTTTCCCGCTGATGGACCCGTAATGAAGAAGCAGACAATTGGCTGG<br>GATAAGTCCTTCGAAAAAATGAGTGTGGCTAAAGAGGTGCTAAGAGGTGATGTGAC<br>TCAGTTTCTTCTGCTCGAAGGAGGTGGTTACCAGAGATGCCGGTTTCACTCCACTTA<br>CAA
**TurboRFP-pBAD**	TCGCAACTCTCTACTGTTTCTCCATACCCGTTTTTTTGGGCTAGAAATAATTTTGTTTA<br>ACTTTAAGAAGGAGATATACATATGCGGGGTTCTCATCATCATCATCATCATGGTAT<br>GGCTAGCATGACTGGTGGACAGCAAATGGGTCGGGATCTGTACGAGAACCTGTACT<br>TCCAGGGCTCGAGCATGAGCGAGCTGATCAAGGAGAACATGCACATGAAGCTGTA<br>CATGGAGGGCACCGTGAACAACCACCACTTCAAGTGCACATCCGAGGGCGAAGGC<br>AAGCCCTACGAGGGCACCCAGACCATGAAGATCAAGGTGGTCGAGGGCGGCCCTC<br>TCCCCTTCGCCTTCGACATCCTGGCTACCAGCTTCATGTACGGCAGCAAAGCCTTC<br>ATCAACCACACCCAGGGCATCCCCGACTTCTTTAAGCAGTCCTTCCCTGAGGGCTT<br>CACATGGGAGAGAATCACCACATACGAAGACGGGGGCGTGCTGACCGCTACCCAG<br>GACACCAGCTTCCAGAACGGCTGCATCATCTACAACGTCAAGATCAACGGGGTGAA<br>CTTCCCATCCAACGGCCCTGTGATGCAGAAGAAAACACGCGGCTGGGAGGCCAAC<br>ACCGAGATGCTGTACCCCGCTGACGGCGGCCTGAGAGGCCACAGCCAGATGGCC<br>CTGAAGCTCGTGGGCGGGGGCTACCTGCACTGCTCCTTCAAGACCACATACAGATC<br>CAAGAAACCCGCTAAGAACCTCAAGATGCCCGGCTTCCACTTCGTGGACCACAGAC<br>TGGAAAGAATCAAGGAGGCCGACAAAGAGACCTACGTCGAGCAGCACGAGATGGC<br>TGTGGCCAAGTACTGCGACCTCCCTAGCAA
**dKatushka-pBAD**	TCTACTGTTTCTCCATACCCGTTTTTTTGGGCTAGAAAtAATTTTGTTTAACTTTAAGA<br>AGGAGATATACATATGCGGGGTTCTCATCATCATCATCATCATGGTATGGCTAGCAT<br>GACTGGTGGACAGCAAATGGGTCGGGATCTGTACGAGAACCTGTACTTCCAGGGCT<br>CGAGCATGGTGGGTGAGGATAGCGTGCTGATCACCGAGAACATGCACATGAAACT<br>GTACATGGAGGGCACCGTGAACGACCACCACTTCAAGTGCACATCCGAGGGCGAA<br>GGCAAGCCCTACGAGGGCACCCAGACCATGAAGATCAAGGTGGTCGAGGGCGGC<br>CCTCTCCCCTTCGCCTTCGACATCCTGGCTACCAGCTTCATGTACGGCAGCAAAAC<br>CTTTATCAACCACACCCAGGGCATCCCCGACTTCTTTAAGCAGTCCTTCCCTGAGG<br>GCTTCACATGGGAGAGGATCACCACATACGAAGACGGGGGCGTGCTGACCGCTAC<br>CCAGGACACCAGCCTCCAGAACGGCTGCCTCATCTACAACGTCAAGATCAACGGG<br>GTGAACTTCCCATCCAACGGCCCTGTGATGCAGAAGAAAACACTCGGCTGGGAGG<br>CCAGCACCGAGATGCTGTACCCCGCTGACAGCGGCCTGAGAGGCCATAGCCAGAT<br>GGCCCTGAAGCTCGTGGGCGGGGGCTACCTGCACTGCTCCCTCAAGACCACATAC<br>AGATCCAAGA
**CarH**	CCAGAAGATCTGGGCACCGGCCTGCTGGAAGCACTGCTGCGCGGTGATCTGGCGG<br>GCGCCGAAGCTCTGTTTCGTCGTGGCCTGCGTTTCTGGGGCCCGGAAGGCGTTCT<br>GGAGCACCTGCTGCTGCCGGTGCTGCGTGAAGTGGGCGAAGCTTGGCACCGTGGT<br>GAAATCGGCGTTGCAGAAGAACACCTGGCGAGCACCTTCCTGCGCGCGCGTCTGC<br>AGGAGCTGCTGGACCTGGCAGGTTTCCCGCCGGGTCCGCCGGTCCTGGTGACTAC<br>GCCGCCGGGCGAACGCCACGAAATCGGTGCGATGCTGGCGGCGTACCATCTGCGT<br>CGTAAGGGCGTCCCGGCGCTGTATCTGGGCCCGGATACTCCGCTGCCGGACCTGC<br>GTGCACTGGCGCGCCGCCTGGGTGCAGGCGCGGTCGTGCTGTCTGCTGTTCTGAG<br>CGAACCGCTGCGTGCTCTGCCTGACGGTGCCCTGAAAGATCTGGCACCGCGTGTT<br>TTCCTGGGCGGCCAGGGCGCAGGCCCGGAAGAGGCACGCCGTCTGGGTGCCGAA<br>TACATGGAAGACCTGAAAGGCCTGGCTGAAGCGCTGTGGCTGCCGCGCGGTCCGG<br>AAAAAGAAGCAATCGGATCCGGTCTCGAG

Table S5. The DNA sequence of MiCy, TurboRFP, dKatushaka, and CarH.
